# Structural Brain Network Reorganization Following Anterior Callosotomy for Colloid Cysts: Connectometry and Graph Analysis Results

**DOI:** 10.3389/fneur.2022.894157

**Published:** 2022-07-18

**Authors:** Marco Ciavarro, Eleonora Grande, Giuseppina Bevacqua, Roberta Morace, Ettore Ambrosini, Luigi Pavone, Giovanni Grillea, Tommaso Vangelista, Vincenzo Esposito

**Affiliations:** ^1^Mediterranean Neurological Institute Neuromed (IRCCS) Neuromed, Pozzilli, Italy; ^2^Department of Neuroscience, Imaging and Clinical Sciences, Gabriele d'Annunzio University, Chieti, Italy; ^3^Department of Translational Medicine, Ferrara University, Ferrara, Italy; ^4^Department of General Psychology, University of Padua, Padua, Italy; ^5^Department of Neuroscience, University of Padua, Padua, Italy; ^6^Padua Neuroscience Center, University of Padua, Padua, Italy; ^7^Department of Human Neurosciences, Sapienza University of Rome, Rome, Italy

**Keywords:** diffusion MRI, anterior callosotomy, white matter, structural connectivity, network topology, graph analysis, colloid cyst

## Abstract

**Introduction:**

The plasticity of the neural circuits after injuries has been extensively investigated over the last decades. Transcallosal microsurgery for lesions affecting the third ventricle offers an interesting opportunity to investigate the whole-brain white matter reorganization occurring after a selective resection of the genu of the corpus callosum (CC).

**Method:**

Diffusion MRI (dMRI) data and neuropsychological testing were collected pre- and postoperatively in six patients with colloid cysts, surgically treated with a transcallosal-transgenual approach. Longitudinal connectometry analysis on dMRI data and graph analysis on structural connectivity matrix were implemented to analyze how white matter pathways and structural network topology reorganize after surgery.

**Results:**

Although a significant worsening in cognitive functions (e.g., executive and memory functioning) at early postoperative, a recovery to the preoperative status was observed at 6 months. Connectometry analysis, beyond the decrease of quantitative anisotropy (QA) near the resection cavity, showed an increase of QA in the body and forceps major CC subregions, as well as in the left intra-hemispheric corticocortical associative fibers. Accordingly, a reorganization of structural network topology was observed between centrality increasing in the left hemisphere nodes together with a rise in connectivity strength among mid and posterior CC subregions and cortical nodes.

**Conclusion:**

A structural reorganization of intra- and inter-hemispheric connective fibers and structural network topology were observed following the resection of the genu of the CC. Beyond the postoperative transient cognitive impairment, it could be argued anterior CC resection does not preclude neural plasticity and may subserve the long-term postoperative cognitive recovery.

## Introduction

Lesions invading the third ventricle include a wide variety of neoplasms and cysts formation typically not affecting the brain parenchyma. Colloid cysts are rare and slow-growing benign lesions, most frequently located in the anterior part of the third ventricle. Due to the increased risk of acute obstructive hydrocephalus, or the development of chronic hydrocephalus-related symptoms (i.e., headache, gait disturbance, and cognitive impairment), surgical excision of these lesions is required in symptomatic cases (1–3). The standard surgical procedures include transcortical, transcallosal, or endoscopic surgical approaches (4). However, when the lesion is close to the neurovascular structures such as bringing veins, arteries, fornix, hypothalamus, or cingulate gyrus, surgical procedures are critical, since healthy brain tissue could be damaged (5) with a high risk for postsurgical cognitive sequelae (6–9). In particular, corticocortical connections such as the corpus callosum (CC) may be damaged in the transcallosal approach as a result of surgical access (9, 10), whereas cortico-subcortical structures, i.e., the fornix, may be affected in all the available surgical approaches as result of surgical manipulation due to the close spatial relation with the lesions (11). Damages to the fornix structures have been widely associated with memory dysfunctions (6, 12–14), caused by the disconnection occurring within the limbic system itself (hippocampus and mammillary bodies) and the connection between the frontal lobes (cingulate gyrus) and the limbic system (thalamus, hippocampus). Otherwise, although the more common cognitive deficits after anterior callosotomy are related to long-term memory impairment, executive dysfunctions, and information exchange between the cerebral hemispheres (e.g., the interhemispheric transfer of motor learning and, to a lesser extent, processing speed) (15), there is still a lack of consensus about permanent cognitive deterioration after anterior body callosal resection (16, 17). Here, we aim at investigating the clinical outcome and the brain functional and structural reorganization after a selective resection of the anterior part of the CC in a cohort of patients who underwent colloidal cyst removal through a new interhemispheric transcallosal surgical approach based on a parallel incision on the genu of CC (18). This surgical approach offers some technical advantages: increasing the line of sight along the anteroposterior axis of the third ventricle, allowing a good exposure and bimanual dissection of the lesion, and facilitating complete lesion removal, but even ensuring a better visualization of the closest crucial anatomical healthy structures.

Diffusion Tensor Imaging (DTI) together with neuropsychological data, were collected pre- and postoperatively to study the clinical outcome and the whole-brain structural reorganization, investigating quantitative anisotropy (QA) index, and also changes in the network topology reorganization after the resection of the anterior CC.

## Materials and Methods

### Study Design and Patient Selection

The study protocol was in accordance with the current STROBE guidelines for cohort studies. A series of six consecutive patients (4 F) were recruited at Neuromed Institute (Pozzilli, Is) during the period between September 2019 and June 2021. Eligible patients were those with a characteristic radiological feature of a cystic lesion in the anterior roof of the third ventricle, confirmed by histological findings, who completed the neuropsychological and radiological follow-up. All the surgical procedures have been performed by the last author (V.E.). All the patients gave their informed consent at the moment of the hospitalization. Data were treated in accordance with the Helsinki declaration.

### MRI Acquisition and Surgical Procedure

All the patients underwent MRI examinations by using a 3-Tesla scanner (GE SignaHDxt, GE Medical Systems, Milwaukee, USA). A 3D T1-weighted (SPGR) structural scan with voxel size = 1 × 1 × 1 mm^3^, repetition time = 7,272 ms, echo time = 300 ms and flip angle = 13° was acquired. dMRI data were collected using a spin-echo echo-planar imaging sequence with 30 diffusion encoding directions with repetition time = 12,200 ms, echo time = 91 ms, slice thickness 3 mm, flip angle 90°, b = 0 and 1,000 s/mm^2^. Both the MRI acquisitions were performed during the week before the surgery and repeated 6 months after surgery (postoperative).

In all the cases, the cyst was located in the anterior part of the third ventricle. The surgical technique requires the access through the non-dominant dura mater, incision in performed on the genu of the CC is and runs parallel to the commissural fibers between the two pericallosal arteries, until the frontal horn of the lateral ventricle is accessed and the right foramen of Monro is identified [see Esposito et al., (18) for details on the surgical procedure].

### Neuropsychological Evaluation

An extensive cognitive evaluation assessing several cognitive domains (i.e., short-term/working memory, long-term memory, attention, praxis abilities, language, abstract reasoning, and executive/frontal cognitive functions) was collected 3 days before the surgery and in two follow-ups: 1 and 6 months after surgery.

The neuropsychological battery included the Mini-Mental State Examination (MMSE); the Frontal Assessment Battery (FAB) for executive functions (both repeated at the second follow-up only); a task of verbal episodic long-term memory (Rey's Auditory Verbal Learning Test, RAVLT and Babcock Story Recall test, BSRT); tasks of spatial (forward Corsi Block-Tapping Test) and verbal (forward and backward Digit Span) short-term/working memory; a task for long-term visuospatial memory (Rey figure recall); abstract reasoning test (Raven's Progressive Matrices ′47, RPM ′47); phonological and semantic verbal fluency; response inhibition (Stroop interference test, SCWT); a task of divided visual attention (Trail Making Test A-B); and constructional praxis (Copy of Rey figures).

Data were corrected for age and educational level and tests parallel versions were adopted to minimize practice effects. Differences among baseline vs. 1 month, 1 vs. 6 months, and baseline vs. 6 months evaluations were analyzed by the Wilcoxon's test for non-parametric testing. The statistical analysis was performed using R studio software 1.3.1. The alpha level was set at 0.05 for statistical significance.

### Image Processing

#### Volumetric Data Segmentation for Ventricular Volume

To establish the presence of ventriculomegaly, a volumetric analysis was performed. The volumes of each brain structure were extracted from 3D T1 scan by using a well-established online software pipeline (http://volbrain.upv.es). This pipeline allows to first improve the quality of the images and analyze and locate them in a common geometric and intensity space and then to perform segmentation at several anatomical levels (19). Each subcortical structure volume was extracted as a relative value, measured in relation to the Intracranial Volume (ICV). The pre- and postoperative volumes of both lateral ventriculi were extracted and analyzed using the Wilcoxon's test.

#### Diffusion MRI Data Processing

The dMRI data were analyzed in DSI Studio software (http://dsi-studio.labsolver.org/). First, the DICOM data of each participant were loaded and renamed to generate the “SRC.gz”, a DSI owner file format containing the raw diffusion weighted images and the metadata associated, namely, image dimension, voxel size and b-table, that are used for reconstruction. DTI were corrected for subject motion and the SRC file was checked to ensure its integrity and quality (20). For each patient and scan (pre- and postoperative), the DTI were reconstructed in a common stereotaxic space by applying the q-space diffeomorphic reconstruction (QSDR) algorithm (21) on the HCP-1021 young adult template, selecting the orientation distribution function (ODF), i.e., the marginal probability of diffusion in a given direction, for mapping the orientation architecture of the tissue. The diffusion sampling length ratio was set to 1.25. The goodness-of-fit was evaluated using the R2 between the warped image and template image. All the patients in this study had *R*^2^ > 0.67. The vector field (fiber orientations) and anisotropy information (the magnitude; “FIB.gz” file) from all the participants were included in the connectometry database and used to conduct fiber tracking analysis. Finally, the patients' scans were paired between baseline scan and follow-up scan for each patient by using the function “Modify a Connectometry Database.”

### Group Connectometry Analysis

Using the DSI studio software, the dMRI connectometry protocol (22) was adopted to identify tracts with significant differences in QA between longitudinal scans, by checking “intercept” as study variables. We tested different levels of *T*-score (2; 2.5; 3) at different significance levels (FDR: 0.05, 0.075 and 0.1) (23) and we found that the results were consistent. Then, by using a deterministic fiber tracking algorithm (24), a *T*-score threshold of 2 was chosen to obtain correlational tractography. The QA values were normalized. We used as terminative region left- and right-cerebellum-white matter and cortex using Freesurfer atlas and as seeding region the whole brain. The tracks were filtered by topology-informed pruning (20) with 1 iteration. A length threshold of 20 voxel distances was used to select tracks. Finally, to estimate the false discovery rate, a total of 2,500 randomized permutations were applied to the group label to obtain the null distribution of the track length.

### Graph Theory: Reorganization in Network Topology

In order to investigate how reorganizations in the tracts drive changes in the network topology, we extracted graph theory measures to estimate changes between pre- and postoperative dMRI scans, by using DSI studio software. Using the “FIB.gz” file for each patient, two different databases were created, specifically for pre- and postoperative scans. The whole-brain fiber tracking of the population average for each database was calculated (10,000,000 seeds). A connectivity matrix was generated using FreesurferSeg atlas (25) with pass region as nodes, the number of fibers passing through a region (i.e., *count*) as metric and a threshold of 0.001. The (weighted) between centrality (BC) metric, a network property considered as an index of the short pathways passing through a specific node, was extracted and computed between scans, by subtracting the preoperative value from the postoperative value as proposed by Taylor et al. (26).

In order to evaluate whether the strength of connection varies between the CC and the nodes of cortical parcellation, a connectivity matrix of connectivity strength was computed. The five subdivisions of CC (posterior, mid-posterior, central, mid-anterior, and anterior, following the DSI studio built-in parcellation on the FreesurferSeg atlas) were considered as nodes and the cortical sites that show changes between pre- and postoperative scans (55 regions for the left hemisphere and 23 regions for the right hemisphere) were extracted by subtracting the preoperative strength value from the postoperative strength value. Then, a 3D graph presentation was obtained from .mat file in DSI Studio and the connectogram was generated with CIRCOS software package (https://mkweb.bcgsc.ca/tableviewer/).

## Results

A total of six consecutive patients (4F; mean age 39.9 years, ranging from 19 to 52 years, mean education was 13.6 years, ranging 8–18 years) who completed neuropsychological and radiological follow-up took part in the study. The transagenual transacallosal surgical approach (18) allowed the gross total resection of colloidal cists in the whole sample. The callosotomy mean was 1.17 ± 0.42 mm (measured on anterior–posterior axis) in all the patients. No disconnection syndrome was registered, and no permanent deficit was reported in all the patients. Although still persisting a post-surgery ventriculomegaly, the volumetric analysis suggested a spontaneous reduction of the preoperative hydrocephalus in five over six patients. In particular, the preoperative lateral ventricle mean value was 6.24% of the total brain volume (±3.21), while the postoperative lateral ventricle mean value was 3.68% (±1.92; *p* > 0.05 between pre- and postventricular volume). No permanent CSF diversion was required.

### Neuropsychological Outcome

Cognitive domains such as working memory, spatial memory, abstract reasoning test, response inhibition, divided visual attention, and constructional praxis showed a slightly worsening 1 month after surgery, with a recovery to the baseline at the second follow-up (Table 1). The screening evaluation and executive function battery (MMSE, FAB) remained stable between the baseline and the follow-up evaluation (*p* > 0.05), suggesting no global cognitive deterioration after surgery. Interestingly, memory performance at RAVLT immediate recall showed a significant improvement between the first and the second follow-up (*p* = 0.031), with a recovery in the short-term memory performance. Although not reaching statistical significance, the post-operative evaluation of RAVLT delayed recall was considerably worse compared to the baseline and the 6-month follow-up evaluation (*p* = 0.062; *p* = 0.059, respectively). Similarly, the BSRT median score collected at 1 month was significantly worse compared with the baseline and the second follow-up (*p* = 0.035; *p* = 0.035, respectively). Moreover, fluency test scores (i.e., phonological and semantic verbal fluency) showed a postoperative recovery: semantic fluency performance at early post-operative was significantly worse than the baseline and the second follow-up (*p* = 0.036; *p* = 0.030, respectively). Finally, the verbal fluency score was significantly worse at 1 month, compared with the 6-month follow-up (*p* = 0.035). Thus, beyond the strong impact of the surgery on the episodic memory and executive functions performances at the early postoperative (<1 month), a cognitive functions recovery was registered at 6 months.

**Table 1:** Neuropsychological evaluations: number of patients with out of normal range (ONR) scores and mean scores among the three evaluations are showed.

**Cognitive domain**	**Neuropsychological test**	**Cut-off**	**Evaluation**	**ONR score (*N*)**	**Mean score**	**1 vs. 6 months (*p* < 0.05)**
Screening	MMSE	23.38	Baseline	0/6	28.37	
				0/6	–	
			6 months	0/6	28.95	
	FAB	13.5	Baseline	1/6	15.64	
				0/6	–	
			6 months	0/6	16.50	
Episodic memory	RAVLT immediate recall	28.5	Baseline	2/6	29.00	
			1 month	4/6	21.63	
			6 months	2/6	31.79	
	RAVLT delayed recall	4.6	Baseline	2/6	5.04	
			1 month	5/6	2.19	
			6 months	3/6	5.26	
	BSRT	8	Baseline	0/6	13.03	
			1 month	3/6	9.01	
			6 months	0/6	16.78	
Working memory	DSF	4.26	Baseline	3/6	4.48	
			1 month	4/6	4.63	
			6 months	2/6	4.64	
	DSB	2.65	Baseline	1/6	3.37	
			1 month	0/6	3.40	
			6 months	0/6	4.35	
Spatial working memory	Corsi block-tapping test	3.5	Baseline	1/6	4.53	
			1 month	2/6	3.87	
			6 months	2/6	4.50	
Visuo-spatial memory	Rey-figure delayed recall	9.47	Baseline	1/6	15.28	
			1 month	0/6	14.48	
			6 months	0/6	19.77	
Abstract reasoning	RPM '47	18.96	Baseline	0/6	28.77	
			1 month	0/6	26.18	
			6 months	0/6	30.65	
Language fluency	Semantic verbal fluency	7.25	Baseline	0/6	14.10	
			1 month	0/6	7.12	
			6 months	0/6	15.52	
	Phonological verbal fluency	10.69	Baseline	1/6	26.73	
			1 month	1/6	20.42	
			6 months	0/6	29.16	
Attention	TMT A	93	Baseline	0/6	37.33	
			1 month	0/6	45.67	
			6 months	0/6	33.00	
	TMT B	282	Baseline	1/6	107.11	
			1 month	0/6	130.44	
			6 months	0/6	103.10	
	TMT B-A	186	Baseline	0/6	69.93	
			1 month	0/6	85.41	
			6 months	0/6	70.24	
Inibhition	SCWT interference time	36.9	Baseline	1/6	36.84	
			1 month	2/6	36.33	
			6 months	1/6	26.80	
	SCWT interference errors	4.2	Baseline	1/6	1.46	
			1 month	1/6	1.80	
			6 months	1/6	1.57	
Constructive apraxia	Rey-figure copy	28.8	Baseline	1/6	32.47	
			1 month	0/6	33.07	
			6 months	0/6	35.48	

*Significative differences were found in: RAVLT immediate recall 1 month vs. 6 months; BSRT baseline vs. 1 month and baseline vs. 6 months; Semantic fluency 1 month vs. baseline and 1 month vs. 6 months; Phonological fluency 1 month vs. 6 months. Out fo Normal Range (ONR)*.

### Postoperative Microstructural White Matter Reorganization and Network Topology

**Figure 1:**
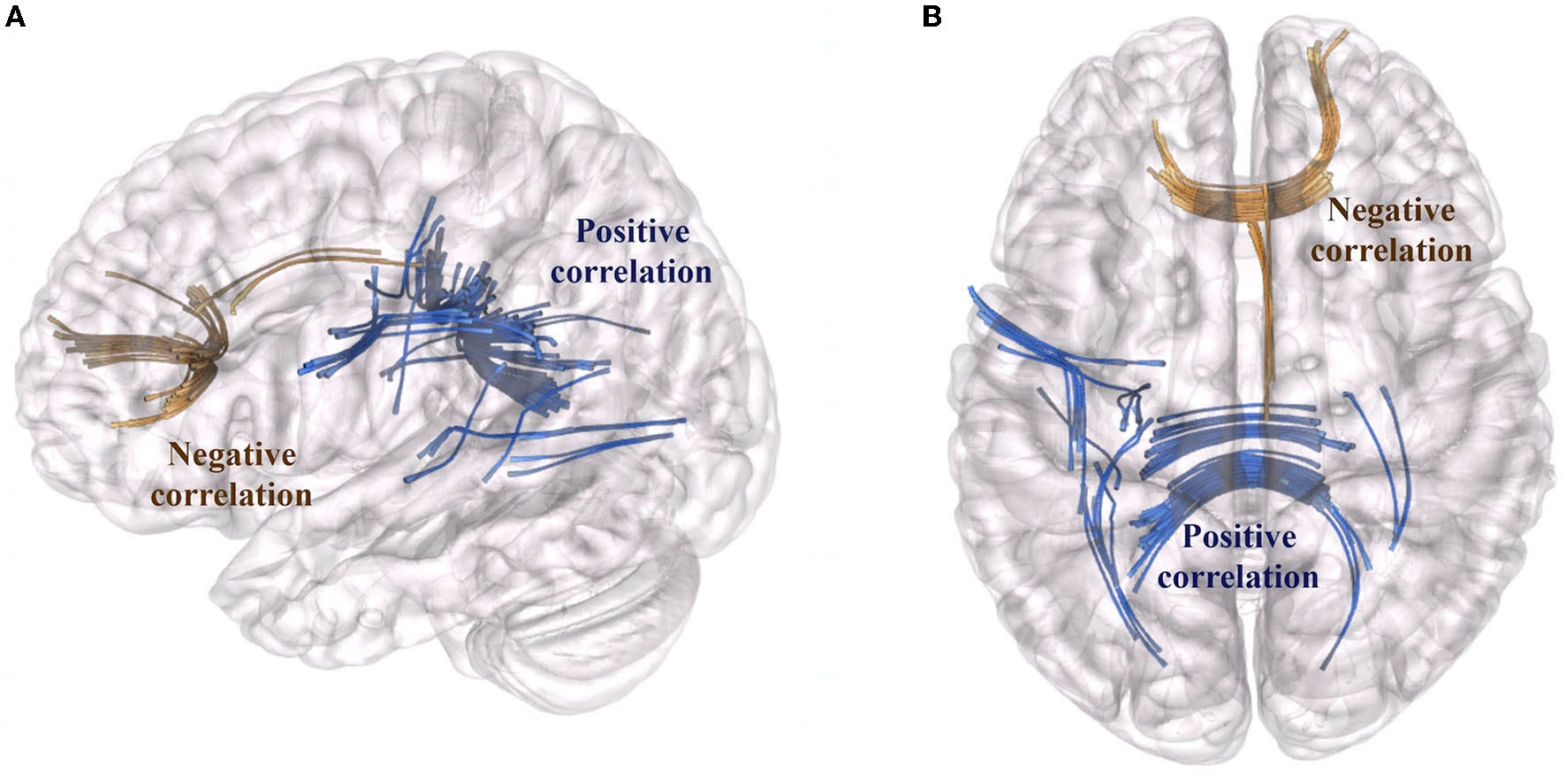
Tract sections with QA changes associated with surgery. Fiber bundles that showed a QA reduction are marked in brown (reticular tract, anterior thalamic radiation, cingulum parolfactory; FDR = 0.011). Fiber bundles that showed an increase of QA (FDR = 0.071) are marked in blue: body, forceps major anterior tapetum of CC and associative bundles of the left hemisphere (frontal aslant tract, optic radiation and superior longitudinal fasciculus). **(A)** Sagittal view, **(B)** Axial view of the brain.

We characterized surgery-induced microstructural changes in white matter and network topology in colloidal cysts patients considering the longitudinal acquisition of dMRI. The results of connectometry analysis showed a significant variation in QA value (FDR < 0.05) between the baseline dMRI and the postoperative scans. Figure 1 showed the subsections of white matter tracts with significant changes in negative and positive QA (FDR < 0.05; *T*-score = 2). First, connectometry study detected widespread surgery-induced changes corresponding to the surgical access (i.e., the genu of CC) and in subsections of the tracts located in the frontal areas of the right hemisphere (reticular tract, anterior thalamic radiation Cingulum Parolfactory, FDR = 0.011). In addition to the previously expected QA reductions, we found QA increased (FDR = 0.071) in the body, forceps major anterior tapetum of CC and in associative bundles of the left hemisphere (frontal Aslant tract, optic radiation and superior longitudinal fasciculus III).

Finally, in order to evaluate whether these changes within the white matter bundle influence the whole-brain network topology, a graph analysis was implemented. Results showed significant changes in BC values in the left hemisphere globally. In particular, BC rises in the left precentral and frontal opercular gyri. It indicates how important the left hemisphere regions are by virtue of being on the shortest paths between other regions, due to the information integrations across the network. By contrast, a decrease in BC was found in the middle and superior frontal gyri bilaterally, with a reduction of their role in passing shortest paths.

**Figure 2:**
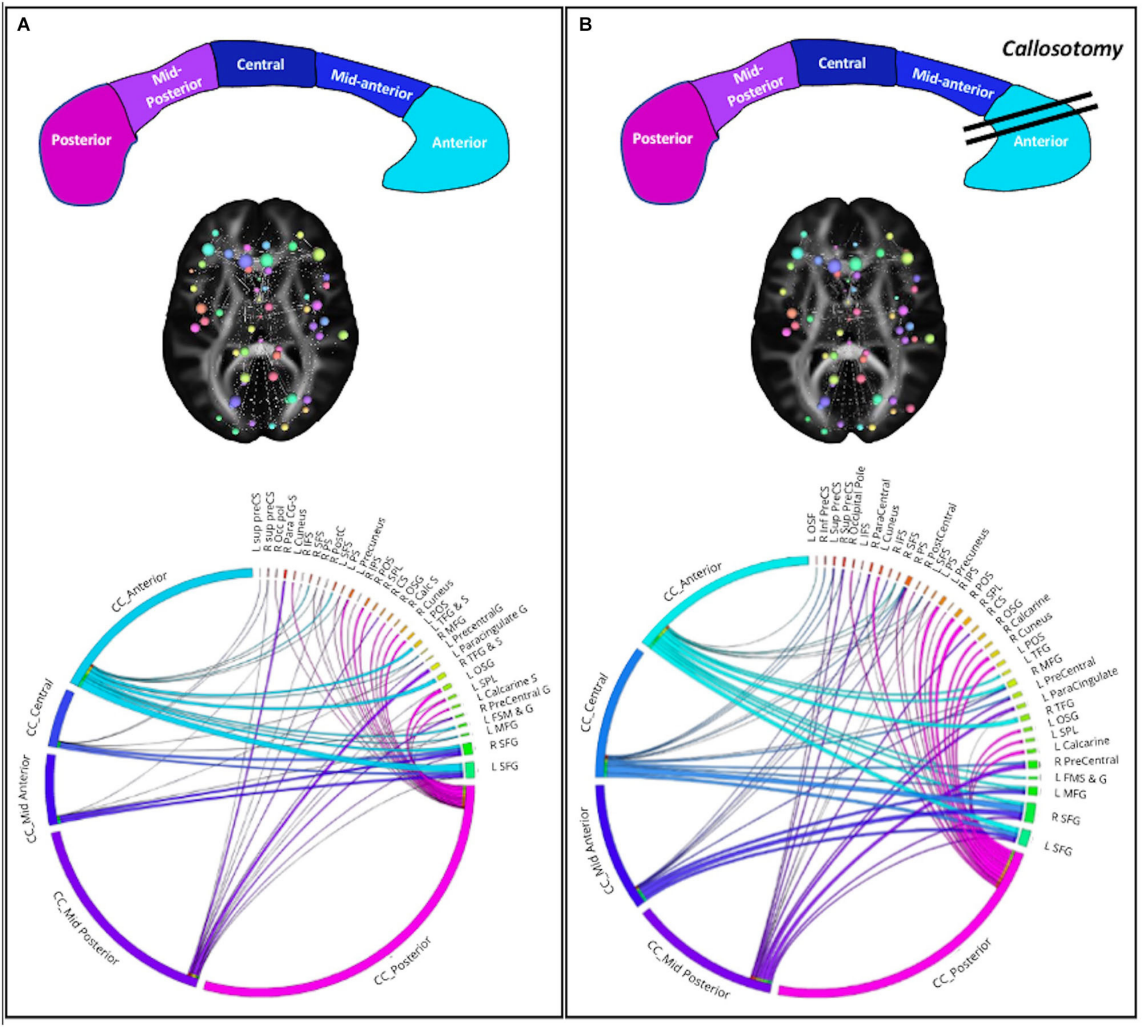
Structural network connectivity changes. **(A)** Preoperative plots; **(B)** postoperative plots. On the top the corpus callosum subsections and surgery. CC subsections are colored as in the CC figure. On the middle the 3D graph visualization of plot connectogram. The cerebral plot shows the brain areas involved in the structural network connectivity changes. On the bottom the Circos plot shows the strength of connectivity among CC subsections and cortical nodes.

Using the connectivity matrix, indicating the connection strength between these nodes and the five sections of CC, we found a postoperative redistribution of fibers connection of middle and superior frontal gyri from anterior CC toward the middle and mid-posterior CC section. On the other hand, considering the connection between the CC and the left precentral region, in which an increase of BC was found, results showed a global increase of these fibers bundles (Figure 2). Notably, we found a global increase of fiber bundles in the mid-anterior and central CC, whereas at the same time a reduction was found in the anterior CC (where callosotomy occurs) as well as in the mid-posterior and posterior CC. Importantly, considering the connection strength between the CC subdivision and the cortical nodes, we found an increase in the number of connections between the mid-anterior and central CC and the cortical nodes, especially the middle and superior frontal gyrus bilaterally. Otherwise, considering the anterior CC, we found a decrease in connection with the right frontomarginal gyrus, whereas the connection with the left frontomarginal gyrus increased considerably. In addition, we found a postoperative reduction of strength between the superior frontal gyrus and anterior CC. Considering the connection between the CC central and mid posterior, we found a postoperative increase of connection between the precentral gyrus, in particular in the right hemisphere. Finally, considering the CC posterior, we found a postoperative increase in connections with the superior parietal and occipital region bilaterally and with the left precuneus.

Taken together, beyond the change in connection strength in the resected area, many regions have a substantial increase in their BC and connectivity strength. It shows a postoperative widely distributed redirection of shortest paths in the network suggesting the existence of many alternative pathways in the brain.

## Discussion

Measures derived from the whole-brain tractography are increasingly being used to characterize the structural connectome of the brain (27–31). This global tractography approach is complementary to the functional connectivity between cortical regions (i.e., resting-state functional connectivity; rsFC) and was used in a growing number of studies investigating the structure-cognition associations in the healthy subjects (32), in developmental and aging studies (33, 34), and also in a range of clinical populations (35–37). The microsurgical removal of lesions affecting the third ventricles (e.g., colloid cysts), although rare, offers a unique opportunity to study how brain plasticity occurs as a consequence of targeted lesioning of CC, and, secondarily, how the structure-cognition relationship could be affected.

Corpus callosum is the major commissural association pathway in the brain, subserving the highest and most complex cognitive functions. It exhibits a topographically functional specialization with different callosal subregions connecting a set of cortical areas: the genu, with small unmyelinated fibers, connects the orbitofrontal and medial prefrontal cortices, whereas mid and posterior regions, with large diameter fibers, connects the temporal, parietal, and occipital regions (38, 39). Although the different origin of the anterior and posterior CC portions seems to correlate with different functional properties, the resection gives rise to different effects with the splenium excision that elicit disconnection syndrome (40), whereas resection of the anterior CC may do not (41). Therefore, the influence of CC lesions on clinical outcome and whether or not the vulnerability of each region of the CC surgical resection impacts the cognitive functions remain unclear.

Here, we investigated the cognitive functions and the structural changes of white matter pathways in a small cohort of patients treated with a recently proposed surgical technique, called the transcallosal transgenual approach (18), which requires the incision of the anterior part of the CC to surgically access the lesion.

To this aim, we performed two complementary analyses to study the whole-brain postoperative white matter reorganization that occurs after a selective resection of the CC. dMRI connectometry analysis based on the QA index and graph analysis of brain structural connectivity was used to identify the specific microstructural and structural changes within subsections of fibers that may subserve the brain connectivity or network topology reorganization (22, 42). Alongside the cognitive recovery registered at the second neuropsychological follow-up, the connectometry analysis of dMRI showed significant changes in white matter structural connectivity. Beyond the loss of fibers close to the resection cavity, an increment in QA was registered in the region of the CC close to the lesion, but not affected by the surgery, as well as in long associative bundles such as Frontal Aslant Tract, Superior Longitudinal Fasciculus and Optic Radiation in the left hemisphere. Accordingly, a considerable change in the structural network connectivity was registered, with an increase of centrality in the left hemisphere nodes, particularly the precentral and the fronto opercular gyrus, suggesting these nodes become more important in the information flow at the whole-brain level.

When occurring a lesion, the shortest path between nodes can change by traversing other areas. Thus, the BC in this region can either increase or decrease following surgery (26). Accordingly, with the significant QA increase in the white matter tracts, most of the regions show a BC increase involving the left hemisphere.

Even the connectivity matrix highlights a new network topology following the surgery, with a strengthening toward the middle and mid-posterior CC section, complementary to the loss of strength in the anterior regions. It could be argued that the selective lesion of the genu of the CC could allow for a brain plastic reorganization at the structural network connectivity level. With the disconnection of some tracts, the shortest paths redirect, leading to an increase in BC in specific left hemisphere nodes.

Moreover, the evidence on the structural white matter and network reorganization after the resection of the genu of the CC might drive brain plasticity and subserve the cognitive recovery to the baseline.

Few publications deal with the neuropsychological outcomes following colloidal cist removal and the role of the surgical excision in the clinical conditions is still debated (43). Here, no sign of disconnection nor attention impairment were registered, neither at early stage, suggesting that the transgenual approach could be safer than other transcallosal approaches. However, tumors involving deep regions, such as the third ventricle, are frequently associated with memory impairment. The critical role of the fornix has been proposed in several researches, suggesting that the more the severity of fornix damage, the more the severity of memory impairment (7, 13, 14, 44). Notably, here no differences in the fornix micro-structures have been reported by the connectometry analysis between pre- and postoperative DTI scans, indicating the integrity of these structures and suggesting transgenual opening is fornix-sparing. Moreover, we registered a good recovery of verbal memory functions at the second follow-up. Although we found a worsening in-memory performance at early postoperative, in line with the literature demonstrating that nearly half of the patients exhibited reduced performance in verbal recall 4 weeks postoperatively (45–50), longer follow-up reported an improvement in cognition (51), as occurred in our patients. Our data can help explain the structural basis of this established cognitive recovery, showing that the microstructural white matter pathway and network topology clearly reorganize following the surgery, and, at the same time, the lesion of the genu of the CC does not prevent this reorganization.

It is important to note that even ventriculomegaly might influence the neuropsychological performance (43, 52), although no patients need CSF diversion, ventriculomegaly was still reported and might be associated with poor cognitive performance still registered at the follow-up.

The present study has some limitations. The small size of the sample and the patients' heterogeneity (e.g., age, ventriculomegaly) reduce its statistical power. Nevertheless, colloid cysts are a rare clinical condition making it hard to reach a homogeneous population. Moreover, despite using other processing methods (i.e., macrostructural changes) [i.e., pre- and postoperative functional connectivity investigation of neuropsychological outcomes (53, 54)] may help explain how callosotomy affects brain reorganization and cognition, actually, pre- and postoperative data collection including both radiological and neuropsychological data are very infrequent in the literature. Finally, we highlight the microstructural changes and structural connectivity graph topology, however, other structural connectivity metrics may play a role in the cognitive recovery, such as fibers' volume.

Further detailed pre- and postoperative neuropsychological studies, or experimental studies investigating more cognitive aspects of interhemispheric transfer of information (e.g., tachistoscopic presentation of stimuli) are required to get deeper on how the surgical approaches, tumor location, and the associated ventriculomegaly affect the cognitive function or structural and functional reorganization. As well, here preliminary results are reported suggesting the importance of safer surgery on patients recovery (55). More researches are necessary to further explain how the white matter reorganization occurs and from which factors are driven.

## Data Availability Statement

The original contributions presented in the study are included in the article/supplementary material, further inquiries can be directed to the corresponding author/s.

## Ethics Statement

The studies involving human participants were reviewed and approved by IRCCS Neuromed (Ethical Approval Code: 11/17 21-12-17). The patients/participants provided their written informed consent to participate in this study.

## Author Contributions

MC and VE: design and conceptualize the study. MC, LP, and GG: data acquisition. MC, EG, GB, and EA: major role in formal analysis. RM, TV, EA, and GB: data curation. MC, EG, and GB: wrote the paper. LP, EA, and VE: revised the manuscript. All authors contributed to the article and approved the submitted version.

## Funding

This work was founded by Neuromed IRCCS, Current Research, by the Italian Ministry of Health.

## Conflict of Interest

The authors declare that the research was conducted in the absence of any commercial or financial relationships that could be construed as a potential conflict of interest.

## Publisher's Note

All claims expressed in this article are solely those of the authors and do not necessarily represent those of their affiliated organizations, or those of the publisher, the editors and the reviewers. Any product that may be evaluated in this article, or claim that may be made by its manufacturer, is not guaranteed or endorsed by the publisher.
